# Impact of feeding strategy after pancreatoduodenectomy on delayed gastric emptying and hospital stay: nationwide study

**DOI:** 10.1093/bjsopen/zraf068

**Published:** 2025-06-13

**Authors:** Tessa E Hendriks, Bo T M Strijbos, Michiel F G Francken, Mahsoem Ali, J Annelie Suurmeijer, Marcel G W Dijkgraaf, Jana S Hopstaken, Kees van Laarhoven, Quintus Molenaar, Vincent E de Meijer, Erwin van der Harst, Marcel den Dulk, Werner Draaisma, Vincent Nieuwenhuijs, Michael F Gerhards, Mike S L Liem, George van der Schelling, Eric Manusama, Ignace de Hingh, Hjalmar van Santvoort, Bas Groot Koerkamp, Olivier R Busch, Bert A Bonsing, Martijn W J Stommel, Marc G Besselink, Dutch Pancreatic Cancer Group (DPCG) and Dutch Institute for Clinical Auditing (DICA), J Haver, E Steenhagen

**Affiliations:** Department of Surgery, Amsterdam UMC, University of Amsterdam, Amsterdam, the Netherlands; Cancer Center Amsterdam, Amsterdam, the Netherlands; Department of Surgery, Leiden University Medical Center, Leiden, the Netherlands; Dutch Institute for Clinical Auditing, Leiden, the Netherlands; Department of Surgery, Radboud University Medical Center, Nijmegen, the Netherlands; Department of Surgery, Amsterdam UMC, University of Amsterdam, Amsterdam, the Netherlands; Cancer Center Amsterdam, Amsterdam, the Netherlands; Department of Surgery, Amsterdam UMC, University of Amsterdam, Amsterdam, the Netherlands; Cancer Center Amsterdam, Amsterdam, the Netherlands; Department of Surgery, Amsterdam UMC, University of Amsterdam, Amsterdam, the Netherlands; Cancer Center Amsterdam, Amsterdam, the Netherlands; Department of Epidemiology and Data Science, Amsterdam UMC, University of Amsterdam, Amsterdam, the Netherlands; Amsterdam Public Health, Methodology, Amsterdam, the Netherlands; Department of Surgery, Radboud University Medical Center, Nijmegen, the Netherlands; Department of Surgery, Radboud University Medical Center, Nijmegen, the Netherlands; Department of Surgery, Regional Academic Cancer Center Utrecht, University Medical Center Utrecht and St Antonius Hospital Nieuwegein, Utrecht, the Netherlands; Department of Surgery, University of Groningen and University Medical Center Groningen, Groningen, the Netherlands; Department of Surgery, Maasstad Hospital, Rotterdam, the Netherlands; Department of Surgery, Maastricht University Medical Center, Maastricht, the Netherlands; NUTRIM-School of Nutrition and Translational Research in Metabolism, Maastricht University, Maastricht, the Netherlands; Department of Surgery, Jeroen Bosch Hospital, ‘s-Hertogenbosch, the Netherlands; Department of Surgery, Isala Clinics, Zwolle, the Netherlands; Department of Surgery, OLVG, Amsterdam, the Netherlands; Department of Surgery, Medisch Spectrum Twente, Enschede, the Netherlands; Department of Surgery, Amphia Hospital, Breda, the Netherlands; Department of Surgery, Medisch Centrum Leeuwarden, Leeuwarden, The Netherlands; Department of Surgery, Catharina Hospital, Eindhoven, the Netherlands; Amsterdam Public Health, Methodology, Amsterdam, the Netherlands; Department of Surgery, Erasmus MC Cancer Institute, Rotterdam, the Netherlands; Department of Surgery, Amsterdam UMC, University of Amsterdam, Amsterdam, the Netherlands; Cancer Center Amsterdam, Amsterdam, the Netherlands; Department of Surgery, Leiden University Medical Center, Leiden, the Netherlands; Department of Surgery, Radboud University Medical Center, Nijmegen, the Netherlands; Department of Surgery, Amsterdam UMC, University of Amsterdam, Amsterdam, the Netherlands; Cancer Center Amsterdam, Amsterdam, the Netherlands

## Abstract

**Background:**

Delayed gastric emptying is a major contributor to prolonged hospital stay following pancreatoduodenectomy. Although enhanced recovery after surgery guidelines recommend unrestricted feeding after pancreatoduodenectomy, nationwide studies evaluating the impact of different feeding strategies after surgery on delayed gastric emptying and length of hospital stay are limited. This study aimed to identify the use and impact of different feeding strategies after pancreatoduodenectomy on delayed gastric emptying and length of hospital stay.

**Methods:**

This nationwide cohort study included consecutive patients after pancreatoduodenectomy from the Dutch Pancreatic Cancer Audit (2021–2023). Primary endpoints were delayed gastric emptying grade B/C and length of hospital stay. Feeding strategies were categorized based on structured interviews with representatives from 15 centres. Multilevel analysis was used to assess associations between feeding strategy, delayed gastric emptying, and length of hospital stay. Predictors of delayed gastric emptying were determined.

**Results:**

Overall, 2354 patients undergoing pancreatoduodenectomy were included, of whom 526 (23%) developed delayed gastric emptying grade B/C. Median length of hospital stay was 13 days longer in patients with delayed gastric emptying (23 *versus* 10 days; *P* < 0.001). Feeding strategies were: unrestricted feeding (3 centres, 637 patients; delayed gastric emptying 18%); step-up feeding (9 centres, 1462 patients; delayed gastric emptying 24%); and artificial feeding (3 centres, 255 patients; delayed gastric emptying 25%). No association was observed between feeding strategy and delayed gastric emptying: step-up *versus* unrestricted feeding (odds ratio 1.14, 95% confidence interval 0.53 to 2.47) and artificial *versus* unrestricted feeding (odds ratio 1.76, 0.65 to 4.73). Similarly, no association was found between feeding strategy and length of hospital stay. The strongest predictor of delayed gastric emptying was pancreatic fistula after surgery (odds ratio 3.16, 2.47 to 4.05).

**Conclusion:**

This study found no significant association between feeding strategy and incidence of delayed gastric emptying or length of hospital stay after pancreatoduodenectomy. Efforts to reduce delayed gastric emptying should focus on reducing pancreatic fistula after surgery.

## Teaser

Delayed gastric emptying significantly prolongs hospital stay after pancreatoduodenectomy; there is much debate as to whether feeding strategy affects this. This nationwide study found no association between feeding strategies (unrestricted, step-up, or artificial feeding) and the rate of delayed gastric emptying or length of hospital stay; however, pancreatic fistula emerged as the strongest predictor of delayed gastric emptying. Efforts to reduce delayed gastric emptying should focus on reducing pancreatic fistula after surgery.

## Introduction

Complications following pancreatoduodenectomy (PD) remain significant^1,2^, with delayed gastric emptying (DGE) being a major contributor to prolonged hospital stay^3,4^. Clinically relevant DGE (grade B/C) is defined by the International Study Group of Pancreatic Surgery (ISGPS)^5^ as the need for nasogastric tube (NGT) drainage for more than 7 days, inability to tolerate solid foods, vomiting, and the need for nasoenteral or parenteral nutrition. Prolonged hospital stay is very common in patients with DGE^6^, and DGE is associated with an estimated €10 000 increase in hospital costs per patient^7^. Effective prophylactic or therapeutic strategies for DGE are currently lacking^5,8^.

The impact of feeding strategies after surgery on the incidence of DGE and subsequent length of hospital stay (LOS) is unclear. The enhanced recovery after surgery (ERAS) guidelines^8^ recommend ‘a normal diet after surgery without restrictions according to tolerance’ (unrestricted diet) following PD. In contrast, many surgeons use a step-up approach for gradually reintroducing oral intake after PD to prevent vomiting and repeated NGT drainage, whereas others^9–12^ advise artificial feeding using nasoenteral or parenteral feeding. A 2022 systematic review^13^, including three retrospective studies and one randomized trial, suggested that an early oral feeding strategy after PD reduced LOS but not the incidence of DGE; however, within ‘oral feeding’, no distinction was made between unrestricted and step-up oral feeding. Consequently, it is unclear which feeding strategy should be advised after PD, concerning the incidence and severity of DGE and LOS. There is still debate as to whether patients should immediately commence an unrestricted diet, or a more stepwise approach or an artificial feeding strategy should be used.

This study aimed to assess the use and impact of feeding strategy on the incidence of DGE grade B/C and LOS after PD.

## Methods

### Study design

This was a nationwide retrospective analysis using data from the Dutch Pancreatic Cancer Audit (DPCA), coordinated by the Dutch Institute for Clinical Auditing and the Dutch Pancreatic Cancer Group (DPCG). The DPCA^14,15^ is a nationwide mandatory registry on pancreatic surgery in the Netherlands that has covered all pancreatic resections since 2014 with an estimated data completeness exceeding 97%. The scientific committee of the DPCG approved the study protocol^16^. According to Dutch law, no ethical approval or informed consent was required, as all data were registered anonymously.

This study included all consecutive patients who underwent PD for all indications and were registered in the DPCA between 1 January 2014 and 31 December 2023. Patients undergoing total pancreatectomy were excluded from the study. During this period, the incidence of DGE was determined per year. Additionally, a structured interview took place with a representative surgeon from each DPCA centre in the Netherlands to assess the protocolized institutional feeding strategy after surgery.

The aim of this study was to evaluate the association between feeding strategy after surgery (for the years 2021–2023, as in these years a protocolized feeding strategy was present which remained unchanged in all participating hospitals) and the incidence of DGE grade B/C and LOS after PD. As secondary outcome, the incidence of DGE grade B/C and feeding strategy per centre in relation to the 3-year PD surgical volume and predictive factors for DGE was assessed. This study was reported in accordance with the STROBE statement^17^.

### Data collection and definitions

Study baseline characteristics consisted of sex, age at the time of surgery, body mass index (BMI), American Society of Anesthesiologists (ASA) grade, and histopathological diagnosis before surgery. Treatment characteristics included neoadjuvant therapy, pylorus resection or preservation, minimally invasive or open surgery, venous or arterial resection, extended resection (in addition to the primary tumour, adjacent structures or organs were removed, such as mesocolon transversum gastric resection), and surgical drain placement. During the study period, neoadjuvant therapy for pancreatic cancer was mainly administered in randomized trials.

Data on feeding strategy and NGT placement (both during and after surgery) were not available in the DPCA. As there were no consistent policies on the use of NGT, this parameter varied per patient; therefore, it was not included in further analysis. Feeding strategy per hospital was determined through from interviews.

Primary outcomes were DGE and LOS per feeding strategy. Outcome parameters were collected during the entire hospital stay and, in the event of earlier discharge, up until 30 days after surgery.

DGE was defined according to the ISGPS^5^. Only clinically relevant DGE (grade B/C) was included. Other outcome parameters included major complications (Clavien–Dindo grade ≥ III)^18^ and pancreatic surgery-related complications, LOS, and readmission within 30 days after discharge. Pancreatic surgery-related complications included postoperative pancreatic fistula (POPF)^19^, postpancreatectomy haemorrhage (PPH)^20^, chyle leakage^21^, and bile leakage^22^, all grade B/C according to the ISGPS or International Study Group for Liver Surgery (ISGLS) criteria. Additionally, DGE was categorized into primary and secondary DGE. Primary DGE refers to the presence of DGE in the absence of other intra-abdominal surgical complications (for example PPH, POPF, bile leakage), and secondary DGE develops in association with intra-abdominal complications after surgery^23,24^. Mortality was defined as in-hospital/30-day mortality (including in-hospital mortality during the entire primary admission or, in case of earlier discharge, up to 30 days).

### Statistical analysis

Baseline patient, tumour, and treatment characteristics are presented using descriptive statistics. Continuous data are presented as median (interquartile range, i.q.r.) or mean(standard deviation), based on data distribution. Categorical variables are presented as counts and proportions. Normally distributed continuous data were compared using one-way ANOVA and non-normally distributed data using the Kruskal–Wallis test. Categorical data were analysed using the χ^2^ test.

Multilevel logistic regression modelling was used to assess the association between different feeding strategies and DGE grade B/C, adjusting for prespecified confounders, to derive odds ratios (ORs) and 95% confidence intervals. Prespecified confounders included sex, age, BMI, ASA grade, Charlson Co-morbidity Index (CCI), diabetes, biliary drainage before surgery, type of surgery (pylorus resection *versus* pylorus-preserving), type of anastomosis (pancreatojejunostomy *versus* pancreatogastrostomy), vascular resection, extended resection, minimally invasive PD, pancreatic adenocarcinoma, POPF, PPH, bile leakage, and centre (as a random intercept). Centre was included in the multilevel model to adjust for differences in DGE incidence and feeding strategies between the included centres. The same regression model was used to assess which variables were most strongly predictive for DGE grade B/C. In this analysis, predictive factors for DGE were ranked by their likelihood ratio χ^2^ value, with a higher value indicating that the variable is more strongly associated with DGE. This approach was used as it is invariant to the scale (continuous/categorical) of the variable, unlike ORs.

Linear mixed models were used to compare LOS between feeding strategies, while adjusting for the same confounders as in the multilevel logistic regression model, with centre as a random intercept, to estimate both the adjusted mean LOS (in days) per feeding strategy and the average (marginal) difference in LOS between feeding strategies.

Missing data were handled using multivariable imputation by chained equations (5 imputations and 10 iterations) with predictive mean matching. Results were pooled across imputed data sets using Rubin's rules.

In a sensitivity analysis, the association between different feeding strategies, DGE grade B/C, and LOS was assessed separately for primary and secondary DGE. In the sensitivity analysis, the same confounders were adjusted for as in the main analysis. Predictors of DGE grade B/C were identified.

Two-sided *P* < 0.050 was considered statistically significant. All analyses were undertaken in R version 4.3.2 (R Foundation for Statistical Computing, Vienna, Austria), using the lme4 and rms packages^25,26^.

## Results

**Fig. 1:**
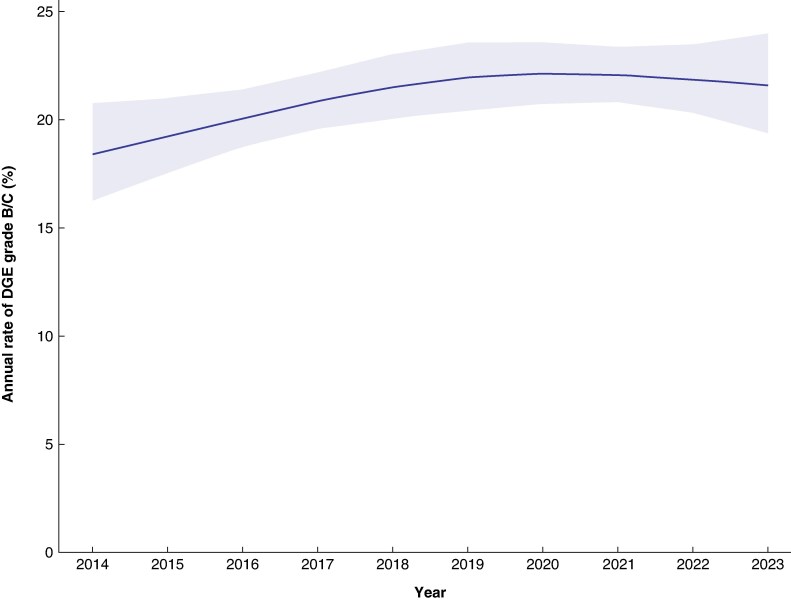
Trend in the annual rate of DGE grade B/C after PD in the Netherlands (2014–2023) Shaded area represents 95% confidence interval. DGE, delayed gastric emptying; PD, pancreatoduodenectomy.

Between 2014 and 2023, the DPCA included 7008 patients who underwent PD, with a mean DGE incidence of 21% (*Fig. 1*). The present study period (2021–2023), in which feeding strategies remained unchanged in all hospitals, included 2354 patients after PD. The mean age was 69 (i.q.r. 61–75) years, 44% of the patients were women, and 42% were diagnosed with pancreatic ductal adenocarcinoma. Most patients underwent open surgery (71%), followed by robot-assisted surgery (26%). The median LOS was 12 (i.q.r. 8–20) days with an in-hospital/30-day mortality rate of 2.7% (*Tables 1–3*). Grades of ISGPS-defined POPF, PPH, chyle leak, and ISGLS-defined bile leakage are reported in *Table S3*.

**Table 1:** Baseline characteristics of patients after PD (2021–2023)

	Overall (*n* = 2354)	Unrestricted feeding (*n* = 637)	Step-up feeding (*n* = 1462)	Artificial feeding (*n* = 255)	P*
**Sex**					0.120
Male	1306 (56%)	346 (54%)	803 (55%)	157 (62%)	
Female	1043 (44%)	291 (46%)	654 (45%)	98 (38%)	
Unknown	5	0	5	0	
**Age (years), median (i.q.r.)**	69 (61–75)	69 (61–75)	69 (61–75)	70 (63–76)	0.416
Unknown	8	0	8	0	
**BMI (kg/m^2^)**					0.765
≤ 25	1418 (62%)	354 (61%)	902 (63%)	162 (64%)	
> 25	858 (38%)	225 (39%)	540 (37%)	93 (36%)	
Unknown	78	58	20	0	
**ASA grade**					0.750
I–II	1425 (62%)	382 (61%)	890 (62%)	153 (60%)	
≥ III	883 (38%)	242 (39%)	539 (38%)	102 (40%)	
Unknown	46	13	33	0	
**CCI score**					0.029
0–1	1325 (63%)	412 (66%)	750 (60%)	163 (65%)	
≥ 2	793 (37%)	210 (34%)	495 (40%)	88 (35%)	
Unknown	236	15	217	4	
**Histological diagnosis**					< 0.001
Pancreatic adenocarcinoma	955 (42%)	254 (40%)	586 (42%)	115 (45%)	
Cholangiocarcinoma	335 (15%)	83 (13%)	222 (16%)	30 (12%)	
Papillary cancer	291 (13%)	63 (9.9%)	188 (13%)	40 (16%)	
Duodenal cancer	142 (6.2%)	57 (9.0%)	71 (5.0%)	14 (5.5%)	
Neuroendocrine neoplasm	97 (4.2%)	31 (4.9%)	61 (4.3%)	5 (2.0%)	
IPMN, SPN, MCN	189 (8.2%)	68 (11%)	106 (7.5%)	15 (5.9%)	
Other	290 (13%)	79 (12%)	175 (12%)	36 (14%)	
Unknown	55	2	53	0	

PD, pancreatoduodenectomy; i.q.r., interquartile range; BMI, body mass index; ASA, American Society of Anesthesiologists; CCI, Charlson Co-morbidity Index; IPMN, intraductal papillary mucinous neoplasm; SPN, solid pseudopapillary neoplasm; MCN, mucinous cystic neoplasm. *Pearson's χ^2^ test or Kruskal–Wallis rank-sum test.

**Table 2:** Operative data and treatment characteristics of patients after PD (2021–2023)

	Overall (*n* = 2354)	Unrestricted feeding (*n* = 637)	Step-up feeding (*n* = 1462)	Artificial feeding (*n* = 255)	*P*‡
**Surgical approach**					< 0.001
Open	1661 (71%)	515 (81%)	988 (68%)	158 (62%)	
Robot-assisted	617 (26%)	120 (19%)	412 (29%)	85 (33%)	
Laparoscopic	56 (2.4%)	1 (0.2%)	43 (3.0%)	12 (4.7%)	
Unknown	20	1	19	0	
**Vascular resection**	411 (19%)	85 (15%)	284 (21%)	42 (16%)	0.006
Unknown	136	57	79	0	
**Extended resection** *	293 (13%)	42 (6.6%)	225 (16%)	26 (10%)	< 0.001
Unknown	15	1	14	0	
**Surgery type**					< 0.001
Pylorus-preserving	801 (34%)	258 (41%)	396 (27%)	147 (58%)	
Pylorus resection	1553 (66%)	379 (59%)	1066 (73%)	108 (42%)	
Unknown	0	0	0	0	
**Drain after surgery**	2155 (95%)	577 (100%)	1325 (92%)	253 (99%)	< 0.001
Unknown	78	59	19	0	
**Neoadjuvant therapy**†	419 (19%)	98 (16%)	282 (20%)	39 (16%)	0.059
Unknown	140	42	84	14	

^*^In addition to the primary tumour, adjacent structures or organs were removed (such as mesocolon transversum, gastric resection). †Neoadjuvant therapy only in patients with pancreatic adenocarcinoma. PD, pancreatoduodenectomy. ‡Pearson's χ^2^ test.

**Table 3:** Characteristics of patients after PD (2021–2023)

	Overall (*n* = 2354)	Unrestricted feeding (*n* = 637)	Step-up feeding (*n* = 1462)	Artificial feeding (*n* = 255)	*P*†
**DGE**					0.007
Grade B/C	526 (23%)	115 (18%)	348 (24%)	63 (25%)	
Unknown	18	1	17	0	
**LOS (days), median (i.q.r.)**	12 (8–20)	12 (8–23)	11 (7–18)	13 (9–20)	< 0.001
Unknown	38	13	25	0	
**Major complications** *	857 (37%)	220 (35%)	573 (40%)	64 (25%)	< 0.001
Unknown	28	6	20	2	
**POPF**					0.424
Grade B/C	489 (22%)	142 (22%)	304 (22%)	43 (18%)	
Unknown	82	1	61	20	
**Bile leakage**					0.021
Grade B/C	144 (6.2%)	53 (8.3%)	80 (5.5%)	11 (4.3%)	
Unknown	21	2	19	0	
**PPH**					0.467
Grade B/C	221 (9.5%)	68 (11%)	130 (9.0%)	23 (9.1%)	
Unknown	24	2	21	1	
**Reintervention**	830 (35%)	208 (33%)	560 (39%)	62 (24%)	< 0.001
Unknown	14	2	12	0	
**Reoperation**	177 (7.6%)	41 (6.5%)	117 (8.1%)	19 (7.5%)	0.420
Unknown	28	3	23	2	
**Readmission**	480 (20%)	112 (18%)	311 (21%)	57 (22%)	0.499
Unknown	150	79	62	9	
**Death**	64 (2.7%)	22 (3.5%)	34 (2.3%)	8 (3.1%)	0.322
Unknown	7	0	7	0	

^*^Clavien–Dindo grade ≥ III^19^. PD, pancreatoduodenectomy; DGE, delayed gastric emptying^5^; LOS, length of hospital stay; i.q.r., interquartile range; POPF, postoperative pancreatic fistula^19^; PPH, postpancreatectomy haemorrhage^20^. †Pearson's χ^2^ test, Kruskal–Wallis rank-sum test or Fisher's exact test.

### Feeding strategies

**Fig. 2:**
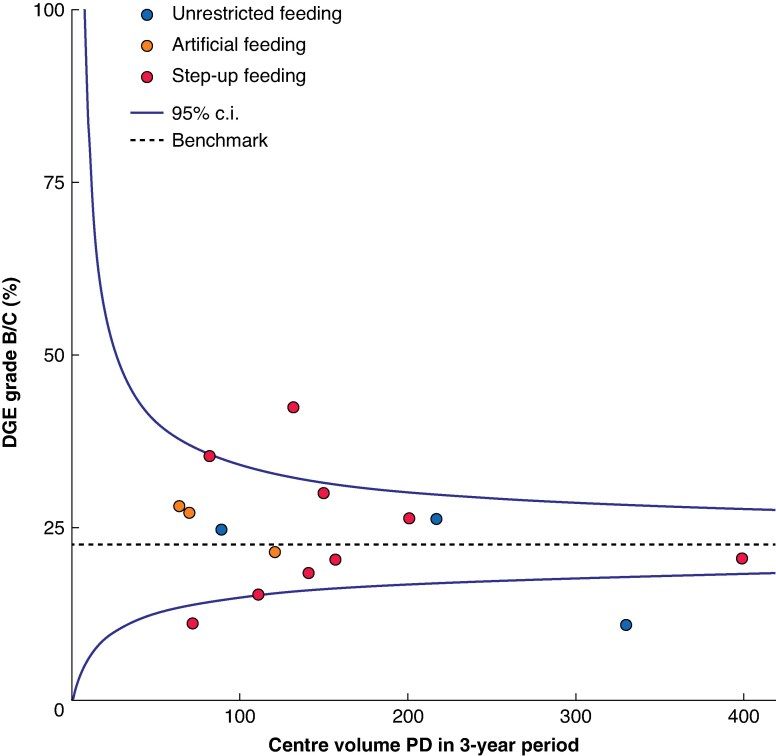
Centre-specific rate of DGE after PD (2021–2023) and routine feeding strategy per centre in relation to 3-year surgical volume DGE, delayed gastric emptying; PD, pancreatoduodenectomy; c.i., confidence interval.

Three distinct feeding strategies were identified: unrestricted feeding (as recommended by ERAS) was used in 637 patients (27%) across 3 centres, with an 18% incidence of DGE grade B/C; step-up feeding, which starts with fluids and gradually builds up towards normal oral intake, was used in 1462 patients (62%) across 9 centres, with a 24% incidence of DGE grade B/C; and artificial feeding, which starts with nasojejunal feeding or total parenteral nutrition, was used in 255 patients (11%) across 3 centres, with a 25% incidence of DGE grade B/C (*P* = 0.007, difference in incidence grade B/C DGE between 3 feeding strategies) (*Table 3* and *Fig. 2*).

### DGE and LOS

Among the 2354 included patients, 526 (23%) developed DGE grade B/C, with 275 (12%) classified as having primary DGE and 251 (11%) secondary DGE. Median LOS was 23 (i.q.r. 16–34) days in patients with DGE grade B/C and 10 (7–15) days in those without DGE (*P* < 0.001) (*Tables 3*, *4*, *S1*, and *S3*).

**Table 4:** LOS of patients with and without clinically relevant DGE

	Overall (*n* = 2336)	No DGE (*n* = 1810)	DGE grade B/C (*n* = 526)	P*
**LOS (days), median (i.q.r.)**	12 (8–20)	10 (7–15)	23 (16–34)	< 0.001
Unknown	33	22	11	

DGE, delayed gastric emptying^5^; LOS, length of hospital stay; i.q.r., interquartile range. *Wilcoxon rank-sum test.

### Multilevel analysis

Compared with the unrestricted feeding strategy, there was no significant association between step-up feeding (OR 1.14, 95% c.i. 0.53 to 2.47) and artificial feeding (OR 1.76, 0.65 to 4.73) and DGE grade B/C. Similarly, there was no association between feeding strategy and LOS (*Table 5*). A *post hoc* sensitivity analysis including additional adjustment for pancreatic duct size and texture did not affect the results materially (*Table S5*).

**Table 5:** Multilevel analysis of feeding strategy in all patients and in those with primary and secondary DGE

	Total* (*n* = 2354)	Primary DGE† (*n* = 1686) (*n* = 275 with DGE)	Secondary DGE‡ (*n* = 668) (*n* = 251 with DGE)
**Feeding strategy, adjusted OR**			
Unrestricted feeding	1.00 (reference)	1.00 (reference)	1.00 (reference)
Step-up feeding	1.14 (0.53, 2.47)	1.18 (0.58, 2.40)	1.32 (0.49, 3.57)
Artificial feeding	1.76 (0.65, 4.73)	1.78 (0.71, 4.50)	1.39 (0.36, 5.41)
**LOS, adjusted MD (days)**§			
Unrestricted feeding	Reference	Reference	Reference
Step-up feeding	−2 (−5, 1)	2 (−6, 9)	−9 (−17, −1)
Artificial feeding (days)	−2 (−6, 2)	2 (−8, 12)	−7 (−24, 10)

Values in parentheses are 95 per cent confidence intervals. *Patients without delayed gastric empyting (DGE)^5^ were included in primary and secondary DGE groups for the multilevel analysis; the exact numbers of patients with DGE are shown. †Patients without any of the following: bile leakage (grade B/C)^23^, postpancreatectomy haemorrhage (PPH) (grade B/C)^21^, POPF (grade B/C)^20^. ‡Patients with any of the following: bile leakage (grade B/C)^23^, PPH (grade B/C)^21^, POPF (grade B/C)^20^. §Multilevel analysis corrected for centre (random effect), sex, age, body mass index, American Society of Anesthesiologists grade, Charlson Co-morbidity Index score, pre-existing diabetes, preoperative biliary drainage, type of surgery (pylorus resection *versus* pylorus-preserving), type of anastomosis (pancreatojejunostomy *versus* pancreatogastrostomy), vascular resection, extended resection (in addition to the primary tumour, adjacent structures or organs were removed, such as mesocolon transversum, gastric resection), minimally invasive pancreatoduodenectomy, pancreatic adenocarcinoma, POPF, PPH grade B/C, and bile leakage grade B/C. OR, odds ratio; LOS, length of hospital stay; MD, mean difference.

### Primary and secondary DGE

In patients with primary DGE, there was no association between step-up feeding (OR 1.18, 95% c.i. 0.58 to 2.40) or artificial feeding (1.78, 0.71 to 4.50) compared with unrestricted feeding for the incidence of DGE. Moreover, LOS did not differ significantly between the step-up and unrestricted feeding strategies, both for patients with primary DGE (22 *versus* 20 days; mean difference 2 (95% c.i. −6 to 9) days) and secondary DGE (32 *versus* 41 days; mean difference −9 (−17 to −1) days). Similarly, no differences in LOS were found between the artificial and unrestricted feeding strategies for primary (22 *versus* 20 days; mean difference 2 (−8 to 12) days) and secondary DGE (33 *versus* 41 days; mean difference−7 (−24 to 10) days) (*Tables 5* and *S2*).

### Predictors

**Fig. 3:**
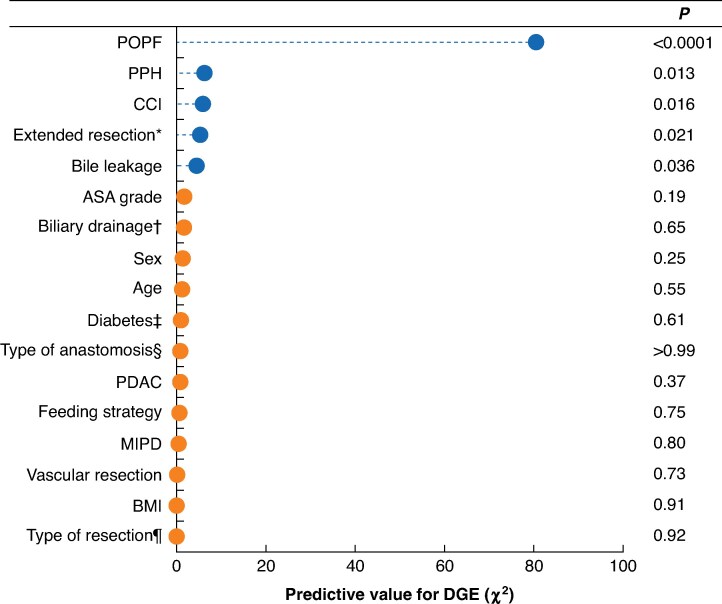
Predictors of DGE grade B/C after PD *In addition to the primary tumour, adjacent structures or organs were removed (such as mesocolon transversum, gastric resection). †Preoperative biliary stenting. ‡Pre-existing diabetes. §Pancreatojejunostomy *versus* pancreatogastrostomy. ¶Pylorus resection *versus* pylorus-preserving. DGE, delayed gastric emptying; PD, pancreatoduodenectomy; POPF, postoperative pancreatic fistula; PPH, postpancreatectomy haemorrhage grade B/C; CCI, Charlson Co-morbidity Index; ASA, American Society of Anesthesiologists; PDAC, pancreatic ductal adenocarcinoma; MIPD, minimally invasive pancreatoduodenectomy; BMI, body mass index.

The following predictors were identified for DGE grade B/C after PD: POPF, CCI, PPH, extended resection, and bile leakage (*P* < 0.050). Among all predictors analysed, POPF had the strongest association with DGE (OR 3.16, 95% c.i. 2.47 to 4.05) (*Fig. 3*).

### *Post hoc* sensitivity analysis—enteral *versus* parenteral nutrition

A *post hoc* sensitivity analysis excluding patients receiving parenteral nutrition showed comparable effects on DGE (*Table S4*). In the unadjusted analyses, a statistically significant difference was found between unrestricted feeding (18% DGE), step-up feeding (24%), and artificial feeding (25%) (*P* = 0.007).

## Discussion

In this nationwide study among 2354 patients undergoing PD, no association was found between feeding strategy after surgery (unrestricted, step-up, and artificial feeding) and the incidence of clinically relevant DGE (grade B/C) and LOS. Notably, despite ERAS guidelines^8^ recommending unrestricted feeding, only one-quarter of patients received this approach; despite this, no meaningful difference in DGE or LOS after PD was observed. Patients who developed DGE experienced a median LOS of 13 days longer than those without DGE. Among the predictors assessed, POPF emerged as the strongest determinant of DGE, along with CCI, PPH, extended resection, and bile leakage.

Limited multicentre studies have assessed the specific association between feeding strategy after PD and DGE, making direct comparisons challenging; however, a meta-analysis^27^ of studies published between 2000 and 2019 evaluated various nutritional approaches, including enteral nutrition after surgery, enteral feeding before surgery, immunonutrition after surgery, and total parenteral nutrition, but did not assess unrestricted oral feeding. Additionally, a more recent single-centre retrospective cohort study^28^ among 428 patients after PD found that an an early oral feeding strategy was associated with a lower incidence of DGE (7.4 *versus* 15%; *P* = 0.005) compared with nasojejunal early enteral nutrition.

The 23% incidence of DGE grade B/C in the present study is higher than the 16–19% reported in previous multicentre studies^4,24,29,30^; however, this discrepancy may be explained by differences in DGE definitions, as not all studies utilized the ISGPS criteria, potentially leading to underestimation in previous reports. Additionally, variations in surgical technique, patient population, or care protocols after surgery may have contributed to differences in incidence of DGE across studies. Previously, audit-based studies^4,29^ using the American College of Surgeons National Surgical Quality Improvement Program reported an incidence of DGE of around 15%, but did not assess feeding strategies after surgery in relation to DGE.

Several studies^31,32^ have explored predictive factors for DGE to identify potential targets for prevention. A Swedish audit-based study^30^ involving 2503 patients after PD also reported POPF as the strongest independent predictor of DGE, which is in line with the present findings. Additionally, the authors found that pylorus-preserving PD (PPPD) and reconstruction with a pancreatogastrostomy were associated with a lower risk of DGE. A pylorus preservation rate of 20% was reported, with a DGE incidence of 19%^30^. In comparison, the present study had a higher pylorus preservation rate of 34%, but a comparable DGE incidence of 23%. Notably, type of resection was not a significant predictor of DGE in the analysis, a finding consistent with previous meta-analysis^33^ showing no clear advantage of pylorus-resecting PD over PPPD in reducing DGE or other complications.

This study represents the largest cohort regarding feeding strategy after PD in relation to DGE. Despite the identified risk factors, the underlying mechanisms of DGE and strategies for its prevention remain unclear. As the primary outcomes did not differ between the three feeding strategies, surgeons should also take secondary downsides into account. For instance, nasojejunal feeding could cause more patient discomfort compared with step-up or unrestricted feeding. Additionally, costs of artificial feeding could be higher compared with those of step-up or unrestricted diet; therefore, the present findings create a new insight into feeding strategies after surgery and could contribute to future changes in national protocols and ERAS guidelines^34^.

Several limitations must be considered when interpreting the results of this study. First, the retrospective nature of the analysis carries inherent risks of bias and confounding, which may affect the validity of findings^35^. To mitigate these concerns, the analysis was adjusted for confounders and centre-level effects. Additionally, interviews revealed that feeding strategies changed over time within hospitals, making it increasingly difficult to recall feeding strategies that were used in the past. To minimize recall bias, the authors restricted the analysis to the most recent 3 years (2021–2023), during which hospitals maintained a single feeding strategy after surgery; however, protocol deviations could have occurred. Second, the categorization of feeding strategies may have introduced some degree of misclassification bias, as some differences could still exist between hospitals in the same category. Third, placement of an NGT for gastric decompression following PD was not recorded in the DPCA, and potentially this could have influenced the incidence and severity of DGE. Fourth, although the results did not show a clinically relevant benefit (such as 10% fewer cases of DGE), it is possible that the strategies do slightly influence the main outcome. Fifth, the present Dutch audit data set does not capture potentially relevant clinical parameters, such as grade A complications, Clavien–Dindo I–II complications, 90-day mortality and baseline nutritional status. Although grade B/C complications are considered clinically relevant, it is possible that grade A complications may still influence recovery after surgery. Furthermore, 90-day mortality might have provided more insight into potential differences between groups, but was not available. Additionally, baseline nutritional status was not captured in the DPCA; therefore, the authors were unable to account for this, although potentially it could still have influenced DGE incidence. Sixth, the artificial feeding category included total parenteral nutrition (1 centre) and enteral feeding (2 centres). A much larger study population would be required to adequately power an analysis capable of detecting clinically meaningful differences^36^. The primary strength of this study lies in its large, nationwide cohort, which provides a comprehensive evaluation of feeding strategies after surgery and their impact on DGE.

DGE remains a challenging complication after PD, influenced by multiple different factors. Future studies should prospectively compare unrestricted feeding (ERAS guidelines) with alternative feeding strategies in randomized trials to provide high-level evidence for clinical guidelines. Additionally, investigating patient-centred outcomes, such as quality of life and functional recovery, in relation to different feeding strategies, could provide a more comprehensive assessment of their benefits. Integrating multimodal approaches, including nutritional support, pharmacological interventions, and enhanced recovery protocols, could further optimize outcomes for patients undergoing PD.

In conclusion, this nationwide study found no evidence to support a specific feeding strategy after PD (unrestricted feeding, step-up feeding, and artificial feeding) to reduce the incidence of DGE grade B/C and LOS. Nevertheless, these findings highlight the need for targeted interventions to reduce DGE, particularly by addressing modifiable risk factors, such as POPF.

## Collaborators

J. Haver, Department of Nutrition and Dietetics, Amsterdam University Medical Center, University of Amsterdam, Amsterdam, the Netherlands. E. Steenhagen, Department of Dietetics, University Medical Center Utrecht, Utrecht, the Netherlands.

## Supplementary Material

zraf068_Supplementary_Data

## Data Availability

Study data are available on request to the corresponding author.
