# Supplementary material for: Impact of feeding strategy after pancreatoduodenectomy on delayed gastric emptying and hospital stay: nationwide study
Source: BJS Open. 2025 Jun 13;9(3):zraf068. doi: 10.1093/bjsopen/zraf068 (PMC12163990; doi:10.1093/bjsopen/zraf068)
Supplement: zraf068_Supplementary_Data [file zraf068_supplementary_data.docx]

**Impact of postoperative feeding strategy after pancreatoduodenectomy on delayed gastric emptying and hospital stay: nationwide study**

Tessa E. Hendriks MD^1,2,3,4,#^, Bo T.M. Strijbos MD^5,#^, Michiel F.G. Francken MD^1,2,#^, Mahsoem Ali BSc^1,2^, J. Annelie Suurmeijer MD PhD^1,2^, Marcel G.W. Dijkgraaf PhD^6,7^, Jana S. Hopstaken MD MA^5^, Kees van Laarhoven MD PhD^5^, Quintus Molenaar MD PhD^8^, Vincent E. de Meijer MD PhD^9^, Erwin van der Harst MD PhD^10^, Marcel den Dulk MD PhD^11, 12^, Werner Draaisma MD PhD^13^, Vincent Nieuwenhuijs MD PhD^14^, Michael F. Gerhards MD PhD^15^, Mike S.L. Liem MD PhD^16^, George van der Schelling MD PhD^17^, E. Manusama MD PhD^18^, Ignace de Hingh MD PhD^19^, Hjalmar van Santvoort^7^, Bas Groot Koerkamp MD, PhD^20^, Olivier R. Busch MD PhD ^1,2^, Bert A. Bonsing MD PhD^3^, Martijn W.J. Stommel MD PhD^5^*, Marc G. Besselink MD MSc PhD^1,2^* for the Dutch Pancreatic Cancer Group (DPCG) and the Dutch Institute for Clinical Auditing (DICA)

^#^shared first, *shared last

**Affiliations:**

^1^Amsterdam UMC, location University of Amsterdam, Department of Surgery, Amsterdam, the Netherlands

^2^Cancer Center Amsterdam, the Netherlands

^3^Department of Surgery, Leiden University Medical Center, Leiden, the Netherlands

^4^Dutch institute for Clinical Auditing, Leiden, The Netherlands

^5^Department of Surgery, Radboud University Medical Center, Nijmegen, the Netherlands

^6^Amsterdam UMC, location University of Amsterdam, Department of Epidemiology and Data Science, Amsterdam, the Netherlands

^7^Amsterdam Public Health, Methodology, Amsterdam, the Netherlands

^8^Department of Surgery, Regional Academic Cancer Center Utrecht, University Medical Center Utrecht and St. Antonius Hospital Nieuwegein, the Netherlands

^9^Department of Surgery, University of Groningen and University Medical Center Groningen, Groningen, the Netherlands

^10^Department of Surgery, Maasstad Hospital, Rotterdam, the Netherlands

^11^Department of Surgery, Maastricht University Medical Center, Maastricht, the Netherlands

^12^NUTRIM-School of Nutrition and Translational Research in Metabolism, Maastricht University, Maastricht, The Netherlands.

^13^Jeroen Bosch Hospital, ‘s-Hertogenbosch, the Netherlands

^14^Isala Clinics, Zwolle, the Netherlands

^15^OLVG, Amsterdam, the Netherlands

^16^Medisch Spectrum Twente, Enschede, the Netherlands

^17^Amphia Hospital, Breda, the Netherlands

^18^Medisch Centrum Leeuwarden, Leeuwarden, the Netherlands

^19^Department of surgery, Catharina Hospital, Eindhoven, the Netherlands

^20^Department of Surgery, Erasmus MC Cancer Institute, Rotterdam, the Netherlands

**Corresponding Author:**
Marc G. Besselink, MD MSc PhD
Amsterdam UMC, University of Amsterdam

Department of Surgery, Cancer Center Amsterdam
De Boelelaan 1117 (ZH-7F), 1081 HV Amsterdam, the Netherlands
Phone: +31 20 4444 00 | Email: [m.g.besselink@amsterdamUMC](mailto:m.g.besselink@amsterdamUMC).nl

**Supplementary Materials - Index**

| **Supplementary Tables** |  |
| --- | --- |
| Table 1: Primary and secondary delayed gastric emptying per feeding strategy | *page 3* |
| Table 2: Supplementary table 2: Multi-level analysis of length of stay per feeding strategy in primary and secondary delayed gastric emptying after pancreatoduodenectomy | *page 4* |
| Table 3: Supplementary table 3: ISGPS complications per grade in patients after pancreatoduodenectomy (2021-2023) | *page 5* |
| Table 4: Post-hoc multi-level analysis of feeding strategy in all patients and in patients with primary and secondary delayed gastric emptying | *page 6* |
| Table 5: Post-hoc multi-level analysis of feeding strategy in all patients including additional adjustment for pancreatic texture and pancreatic duct diameter  Study questionnaire | *page 7*  *page 8* |
| **References** | *page 9* |

**Supplementary Tables**

**Supplementary table 1: Primary and secondary delayed gastric emptying per feeding strategy**

|  | **Total DGE**  n = 526* | **Unrestricted**  **feeding**  n = 115 | **Step-up**  **feeding**  n = 348 | **Artificial**  **feeding**  n = 63 | **P value*** |
| --- | --- | --- | --- | --- | --- |
| **DGE type** |  |  |  |  | 0.065 |
| **Primary** | 275 (52) | 54 (47) | 180 (52) | 41 (65) |  |
| **Secondary** | 251 (48) | 61 (53) | 168 (48) | 22 (35) |  |

*DGE: delayed gastric emptying (defined by ISGPS^1^)*

**Fisher’s exact test*

*Note: variables as number (percentage)*

**Supplementary table 2: Multi-level analysis of length of stay per feeding strategy in primary and secondary delayed gastric emptying after pancreatoduodenectomy**

|  | **Total**^§^  n = 2,354 | **Primary DGE^#^**  n = 1,686  (275 patients had DGE) | **Secondary DGE^†^**  n = 668  (251 patients had DGE) |
| --- | --- | --- | --- |
| **Strategy** | Adj mean*, days  (95% CI) | Adj mean*, days  (95% CI) | Adj mean*, days  (95% CI) |
| **Unrestricted feeding** | 18 (15 – 22) | 20 (13 – 27) | 41 (34 – 47) |
| **Step-up feeding** | 17 (14 – 19) | 22 (18 – 26) | 32 (28 – 36) |
| **Artificial feeding** | 16 (12 – 20) | 22 (15 – 29) | 33 (19 – 47) |

*Adj mean: adjusted mean, DGE: delayed gastric emptying (defined by ISGPS^1^), CI: Confidence Interval.*

**Multi-level analysis corrected for center (random effect), sex, age, Body Mass Index, ASA score, Charlson Comorbidity Index, preexistent diabetes, preoperative biliary drainage, type of surgery (pylorus resection vs pylorus preserving), type of anastomosis (pancreatojejunostomy vs pancreatogastrostomy), vascular resection, extended resection (in addition to the primary tumor, adjacent structures or organs were removed: e.g. mesocolon transversum, gastric resection), minimally invasive pancreatoduodenectomy, pancreatic adenocarcinoma, postoperative pancreatic fistula, post pancreatectomy hemorrhage grade B/C, and bile leak grade B/C.*

*^#^Patients without any of the following: bile leak (grade B/C)^23^, post pancreatectomy hemorrhage (grade B/C)^21^, post operative pancreatic fistula (grade B/C^20^).*

*^†^Patients with any of the following: bile leak (grade B/C)^23^, post pancreatectomy hemorrhage (grade B/C)^21^, post operative pancreatic fistula (grade B/C^20^).*

*^§^Patients without were included in primary and secondary DGE groups for the multi-level analysis, the exact number of patients with DGE are shown.*

**Supplementary table 3: ISGPS and ISGLS complications per grade in patients after pancreatoduodenectomy (2021-2023**)

|  | **Overall**  n = 2,354 | **Unrestricted**  **feeding**  n = 637  (27%) | **Step-up**  **feeding**  n = 1,462  (62%) | **Artificial**  **feeding**  n = 255  (11%) | **P value**^1^ |
| --- | --- | --- | --- | --- | --- |
| **DGE** |  |  |  |  | 0.007* |
| **Grade B/C** | **526 (23)** | **115 (18)** | **348 (24)** | **63 (25)** |  |
| *Unknown* | 18 | 1 | 17 | 0 |  |
| - *Grade B* | *278 (12%)* | *52 (8.2%)* | *190 (13%)* | *36 (14%)* |  |
| - *Grade C* | *248 (11%)* | *63 (9.9%)* | *158 (11%)* | *27 (11%)* |  |
| **POPF** |  |  |  |  | 0.424 |
| *Grade B/C* | 489 (22) | 142 (22) | 304 (22) | 43 (18) |  |
| *Unknown* | 82 | 1 | 61 | 20 |  |
| - *Grade B* | 456 (20%) | 131 (21%) | 283 (20%) | 42 (18%) |  |
| - *Grade C* | 33 (1.5%) | 11 (1.7%) | 21 (1.5%) | 1 (0.4%) |  |
| **Bile leakage** |  |  |  |  | 0.021* |
| *Grade B/C* | 144 (6.2) | 53 (8.3) | 80 (5.5) | 11 (4.3) |  |
| *Unknown* | 21 | 2 | 19 | 0 |  |
| - *Grade B* | 110 (4.7%) | 44 (6.9%) | 60 (4.2) | 6 (2.4%) |  |
| - *Grade C* | 34 (1.5%) | 9 (1.4%) | 20 (1.4) | 5 (2.0%) |  |
| **PPH** |  |  |  |  | 0.467 |
| *Grade B/C* | 221 (9.5) | 68 (11) | 130 (9.0) | 23 (9.1) |  |
| *Unknown* | 24 | 2 | 21 | 1 |  |
| - *Grade B* | 109 (4.7%) | 42 (6.6%) | 58 (4.0%) | 9 (3.5%) |  |
| - *Grade C* | 112 (4.8%) | 26 (4.1%) | 72 (5.0%) | 14 (5.5%) |  |
| **Chyle leak** |  |  |  |  | 0.033* |
| *Grade B/C* | 140 (12%) | 30 (9.4%) | 10 (6.9%) | 100 (13%) |  |
| *Unknown* | 804 | 190 | 107 | 507 |  |
| - *Grade A* | 330 (21%) | 100 (22%) | 32 (22%) | 198 (21%) |  |
| - *Grade B* | 136 (8.8%) | 28 (6.3%) | 10 (6.8%) | 98 (10%) |  |
| - *Grade C* | 4 (0.3%) | 2 (0.4%) | 0 (0%) | 2 (0.2%) |  |
| *^1^Pearson’s Chi-squared test; Kruskal-Wallis rank sum test,* *Fisher’s exact test. *Indicates statistical significance.*  *DGE: delayed gastric emptying^5^, POPF: postoperative pancreatic fistula^20^, PPH: post pancreatectomy hemorrhage^21^* | | | | | |

**Supplementary table 4: Post-hoc multi-level analysis of feeding strategy in all patients and in patients with primary and secondary delayed gastric emptying**

|  | **Total**^§^  n = 2,354 | | **Primary DGE^#^**  n = 1,686  (275 patients had DGE) | | **Secondary DGE^†^**  n = 668  (251 patients had DGE) | |
| --- | --- | --- | --- | --- | --- | --- |
| **Feeding strategy** | aOR (95% CI) | | aOR (95% CI) | | aOR (95% CI) | |
| Unrestricted feeding | Ref. | - | Ref. | - | Ref. | - |
| TPN | 2.51 | (0.64 to 9.78) | 2.51 | (0.64 to 9.78) | 4.00 | (0.65 to 24.56) |
| Enteral feeding | 1.50 | (0.52 to 4.33) | 1.97 | (0.72 to 5.27) | 0.73 | (0.16 to 3.28) |
| **Length of stay** | aMD* (95%CI) | | aMD* (95%CI) | | aMD* (95%CI) | |
| Unrestricted feeding | Ref. | - | Ref. | - | Ref. | - |
| TPN | –2 | (–8 to 4) days | 1 | (–5 to 7) days | –11 | (–22 to -0) days |
| Enteral feeding | –2 | (–6 to 3) days | 3 | (–1 to 8) days | –17 | (–26 to 8) days |
| *aOR: adjusted Odds Ratio, aMD: adjusted Mean Difference. DGE: delayed gastric emptying (defined by ISGPS^5^), CI: Confidence Interval, TPN: total parenteral nutrition*  **Multi-level analysis corrected for center (random effect), sex, age, Body Mass Index, ASA score, Charlson Comorbidity Index, preexistent diabetes, preoperative biliary drainage, type of surgery (pylorus resection vs pylorus preserving), type of anastomosis (pancreatojejunostomy vs pancreatogastrostomy), vascular resection, extended resection (in addition to the primary tumor, adjacent structures or organs were removed: e.g. mesocolon transversum, gastric resection), minimally invasive pancreatoduodenectomy, pancreatic adenocarcinoma, postoperative pancreatic fistula, post pancreatectomy hemorrhage grade B/C, and bile leak grade B/C.*  *^#^Patients without any of the following: bile leak (grade B/C)^23^, post pancreatectomy hemorrhage (grade B/C)^21^, post operative pancreatic fistula (grade B/C^20^).*  *^†^Patients with any of the following: bile leak (grade B/C)^23^, post pancreatectomy hemorrhage (grade B/C)^21^, post operative pancreatic fistula (grade B/C^20^).*  ^§^*Patients without DGE were included in primary and secondary DGE groups for the post-hoc multi-level analysis, the exact number of patients with DGE are shown.* | | | | | | |

**Supplementary table 5: Post-hoc multi-level analysis of feeding strategy in all patients including additional adjustment for pancreatic texture and pancreatic duct diameter**

|  | **Total**^§^  n = 2,354 | |
| --- | --- | --- |
| **Feeding strategy** | aOR (95% CI) | |
| Unrestricted feeding | Ref. | - |
| Step-up feeding | 1.49 | (0.64 to 3.48) |
| Artificial feeding | 2.26 | (0.73 to 6.99) |
| *aOR: adjusted Odds Ratio, CI: confidence interval, DGE: delayed gastric emptying.*  **Multi-level analysis corrected for center (random effect), sex, age, Body Mass Index, ASA score, Charlson Comorbidity Index, preexistent diabetes, preoperative biliary drainage, type of surgery (pylorus resection vs pylorus preserving), type of anastomosis (pancreatojejunostomy vs pancreatogastrostomy), vascular resection, extended resection (in addition to the primary tumor, adjacent structures or organs were removed: e.g. mesocolon transversum, gastric resection), minimally invasive pancreatoduodenectomy, pancreatic adenocarcinoma, postoperative pancreatic fistula, post pancreatectomy hemorrhage grade B/C, and bile leak grade B/C, pancreatic texture, and pancreatic duct diameter.*  ^§^*total number of patients including patients without DGE.* | | |

**Impact of postoperative feeding strategy after pancreatoduodenectomy on delayed gastric emptying and hospital stay: a nationwide study - Surgeon questionnaire**

**Nutritional strategy**

1. Nutritional strategy
   1. Are nasogastric feeding tubes used? If yes, specify the following: timing of placement and indication (structurally/regularly/occasionally/never)
   2. Are nasojejunal (deep) feeding tubes used? If yes, specify the following: timing of placement and indication (structurally/regularly/occasionally/never)
   3. Is parenteral nutrition used? If yes, specify the timing of initiation and indication (structurally/regularly/occasionally/never)
   4. Are transabdominal feeding tubes (e.g., feeding jejunostomy) routinely placed? If yes, please specify the timing of placement and indication
   5. How is postoperative feeding initiated? If protocolized, we request a copy of this protocol.
2. Have there been any changes in the indication for enteral/parenteral nutrition or in the protocol for initiating nutrition between 2014 and 2022? If so, what were they and when (month and year)?

**Policy when enteral feeding is not feasible**

1. What is the policy when enteral feeding is not feasible? Please specify the use and indications for parenteral nutrition
2. Have there been any changes in the indication and use of deep feeding tubes and/or parenteral nutrition between 2021 and 2023? If so, what were they and when (month and year)?

**Nutritional strategy categorization**

We classify centers based on the nutritional strategy used immediately postoperatively. There are three categories:

1. **Unrestricted feeding:** patients are allowed a normal diet after surgery without restrictions according to tolerance as part of the standardized hospital protocol. No standard artificial feeding (parenteral or naso-gastic tube feeding) is used.
2. **Step-up feeding:** The postoperative protocol, prescribes a slow build op to normal feeding after surgery. Patients start with fluids and gradually build up toward normal oral intake. No solid intake at postoperative day 1.
3. **Artificial feeding:** Each patient receives artificial nutrition as a support or as a total replacement of the normal oral diet in the postoperative phase. This means either a standized immediate start with nasojejunal feeding tube or total parenteral nutrition.

In which category does your center fall regarding the immediate postoperative nutritional strategy after pancreatoduodenectomy? Category 1, 2, or 3? This concerns the nutritional policy in the period 2021-2023.

**References**

1. Wente MN, Bassi C, Dervenis C, Fingerhut A, Gouma DJ, Izbicki JR, et al. Delayed gastric emptying (DGE) after pancreatic surgery: a suggested definition by the International Study Group of Pancreatic Surgery (ISGPS). Surgery. United States; 2007 Nov; 142: 761–768.
